# Patient conversations that matter: assessing the role of TARGET TYI leaflets for respiratory care in England’s community pharmacies

**DOI:** 10.1093/jacamr/dlaf196

**Published:** 2025-11-18

**Authors:** Sejal Parekh, Lingqian Xu, Kieran Hand, Diane Ashiru-Oredope, Donna M Lecky

**Affiliations:** Primary Care Strategy and NHS Contracts Group, Primary Care, Community Services and Personalised Care Directorate, NHS England, London SE1 8UG, UK; Primary Care Strategy and NHS Contracts Group, Primary Care, Community Services and Personalised Care Directorate, NHS England, London SE1 8UG, UK; AMR Programme, Medical Directorate, NHS England, London SE1 8UG, UK; HCAI, Fungal, AMR, AMU & Sepsis Division, UK Health Security Agency, London SW1P 3HX, UK; HCAI, Fungal, AMR, AMU & Sepsis Division, UK Health Security Agency, London SW1P 3HX, UK

## Abstract

**Objectives:**

The TARGET Treating Your Infection (TYI-RTI) leaflets are designed to support healthcare professionals in delivering tailored conversations with patients about respiratory tract infection (RTI) symptoms. This study evaluated their use across community pharmacies in England through the Pharmacy Quality Scheme (PQS).

**Methods:**

In 2022–23 and 2023–24, the PQS incentivized community pharmacies to use TYI-RTI leaflets for patients presenting with upper RTIs during a 4 week period each year.

**Results:**

Across the 2 years, 225 615 patients were reviewed using TYI-RTI leaflets. A total of 7525 pharmacies participated in at least 1 year, with 7407 taking part in both. The proportion of patients requiring escalation to the pharmacist fell from 23% (26 678) in 2022–23 to 15% (16 417) in 2023–24. Approximately one-third of patients were managed entirely by the wider pharmacy team. Signposting to another healthcare provider also declined, from 12% (13 248) to 7% (8250; *P* < 0.001), with immediate escalation reducing from 6% (6859) to 4% (4075; *P* < 0.001). Most patients were offered over-the-counter (OTC) remedies—85% (97 551) in 2022–23 and 89% (98 343) in 2023–24 (*P* < 0.001)—with high acceptance rates (80% and 84%, respectively). Self-care advice was provided to almost all patients [95% (109 474) in 2022–23; 97% (106 770) in 2023–24; *P* < 0.001]. Around one-third received additional written information in both years [31% (35 624) and 31% (34 242)].

**Conclusions:**

Community pharmacies successfully used TYI-RTI leaflets to support patient self-care, reassurance and safety-netting, reducing the need for escalation. The study highlights the role of TYI-RTI in optimizing pharmacy-led RTI management and reducing pressure on wider healthcare services. Incentivization was an effective tool for improving AMS in community pharmacy at pace.

## Introduction

The greatest proportion of antibiotics for human use are prescribed in the primary healthcare sector for the treatment of two common infections—respiratory tract infection (RTI) and urinary tract infection (UTI).^1^ Barriers to appropriate antibiotic use include patients demanding antibiotics, the perception that patients expect antibiotics, and prescribing antibiotics to save time rather than explain why antibiotics are not needed.^2–4^

Upper RTIs (URTIs) are primarily caused by viruses or bacteria infecting the nose, sinuses, pharynx and larynx.^5^ The infections present acutely, when patients can experience laryngitis, pharyngitis, nasopharyngitis and rhinitis whilst complaining of symptoms such as a cough, sore throat or sinusitis.^6^ As the majority of the URTIs are viral in nature, there is limited evidence that antibiotics are effective in treating these conditions in adults and children.^7^ However, in England, it is common for patients to attend GP appointments where there is a 50%–100% chance of receiving an antibiotic such as amoxicillin.^8,9^

Community pharmacy teams are trusted members of the healthcare workforce, with community pharmacies being open at unsocial hours and at weekends.^10^ It is common for patients to seek over-the-counter (OTC) remedies for symptom relief. Some of these medications are classified as pharmacy-only or ‘P’ medicines and can only be bought under a pharmacist’s supervision. Pharmacy teams utilize patient symptoms and past medical history to assess which product(s) are suitable. These conversations provide an opportunity to offer symptom management advice to the patient.

Outcome 2 of the UK’s 5-year National Action Plan on AMR states: ‘By 2029, the public are empowered and equipped to engage on and help address AMR through an improved understanding of the scale and nature of the risk, and the individual behavioural actions that can be taken to address it’.^11^

The UK Health Security Agency (UKHSA)-led ‘Treat Antibiotics Responsibly Guidance, Education and Tools’ (TARGET) Toolkit consists of a range of evidence-based tools to support and promote antimicrobial stewardship (AMS) for healthcare professionals and patients.^12^

**Figure 1.**
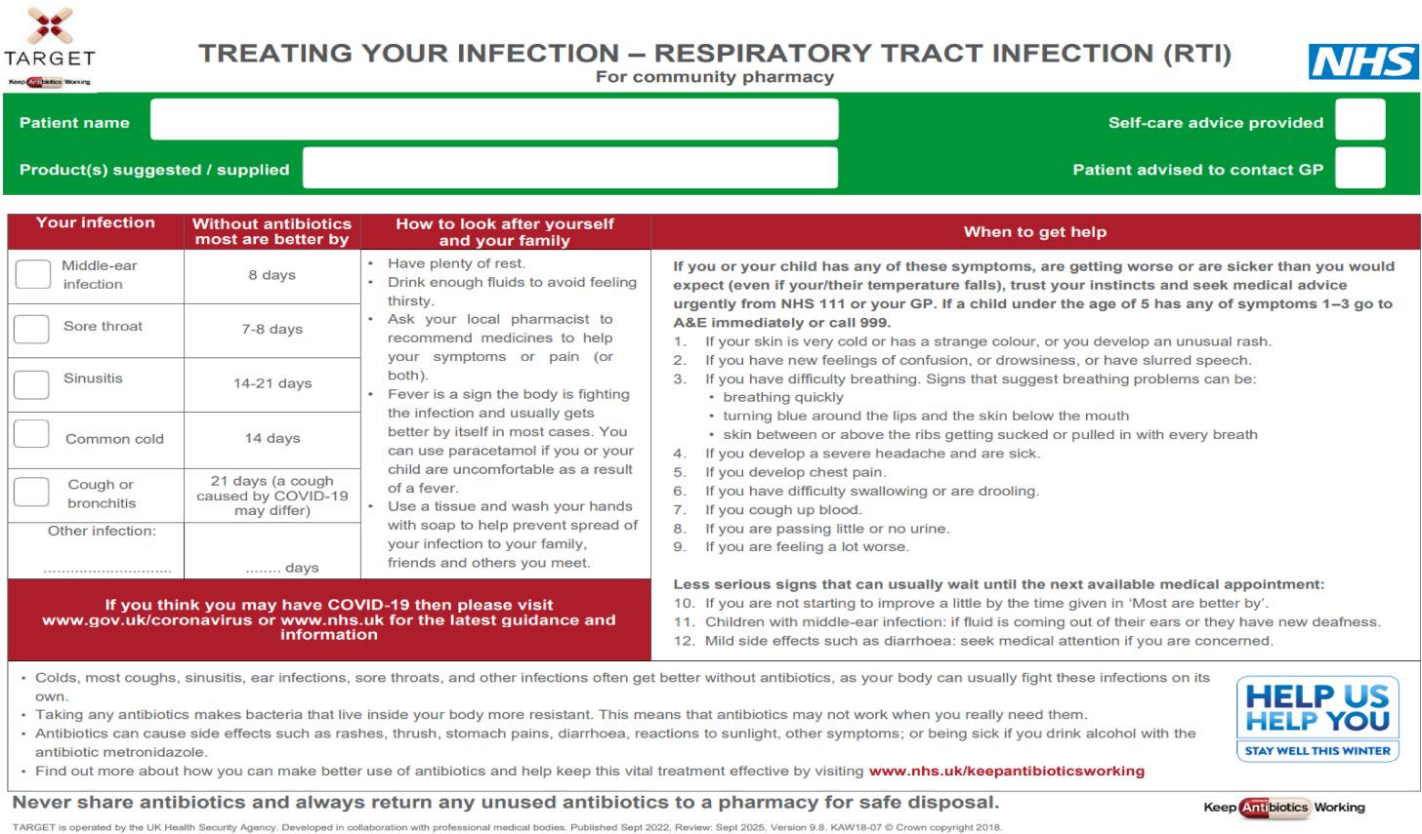
TARGET: TYI-RTI leaflet.

The TARGET Treating Your Infection leaflets for RTI (TYI-RTI) are designed for healthcare professionals to have structured discussions with patients about their symptoms (Figure 1).^13^ They can be tailored and personalized to patients, to explain the average duration of their ailment, self-care and management of their symptoms with safety-netting, advising when patients should seek further advice and reconsult with a healthcare professional.^12,14^

Since 2020, England’s community pharmacy teams have received AMS and infection prevention and control (IPC) training, as well as being incentivized to use the TARGET tools through the Pharmacy Quality Scheme (PQS).^15–17^ The PQS is designed to reward community pharmacy contractors for delivering three quality dimensions, specifically clinical effectiveness, patient safety and patient experience.^18^ In 2023–24, the PQS re-incentivized all three TARGET Tools—the TARGET Antibiotic Checklist, and the TARGET TYI leaflets for both RTI and UTI.^19^ This study evaluates the 2 years 2022–23 and 2023–24 and the impact of the TARGET TYI-RTI leaflets in community pharmacy, comparing the uptake of the leaflets, staff groups using the leaflets, and numbers of patients adequately managed in the pharmacy, including identifying triggers for escalation and OTC recommendations.

## Materials and methods

The detailed methodology has been outlined previously, where both TARGET TYI leaflets for UTI and RTI were incentivized simultaneously.^17^

### Study design

This is a service evaluation of the scaling of the use of the TARGET TYI-RTI leaflet in community pharmacies via the national incentive scheme. In this structured observational study, researchers assessed the outcomes of how community pharmacists implemented the use of the leaflets within the AMS domain of the PQS criteria.

### Setting and participants

There were 11 051 and 11 043 registered community pharmacies in England in March 2023 and March 2024, respectively.^20^ Community pharmacy teams were asked to record data from walk-in patients requesting advice for the management of symptoms of RTIs. Community pharmacy teams consist of registered pharmacy professionals (pharmacists and technicians) and non-registered pharmacy staff (trainee pharmacists, trainee technicians, dispensary staff and medicines counter assistants), all being patient-facing staff who provide advice on medicines.

Any patient with RTI symptoms was eligible for inclusion. The data collected were subdivided by age, specifically patients under 5 years of age, over 5 (up to 17 years) and adults (18 years and over).^21^ The follow-up of the outcome of patients signposted to other healthcare settings was outside the scope of this study.

### Data collection

Pharmacy teams were required to submit the data collected via the Manage Your Service (MYS) software portal by either the date of their PQS declaration or no later than 31 March 2023 or 31 March 2024. They could complete the data collection from the launch of the scheme on 10 October 2022 to its closure on 31 March 2023 in PQS 2022–22, and from 1 June 2023 to 31 March 2024 for PQS 2023–24. The NHS Business Services Authority (NHSBSA) produced a digital version of the questions from the TARGET TYI leaflets, which pharmacy teams were required to input for data collection. These were made available to all pharmacy contractors via the MYS portal for data submission. A single form submission was required per patient. Pharmacy teams could complete the data collection at any timepoint during the duration of the PQS.

The results from the PQS 2022–23 TARGET TYI-RTI data were published on the NHS England website, with findings and recommendations that community pharmacy teams needed to implement into day-to-day practice.^22^ The use of the TARGET TYI-RTI leaflets was re-incentivized in the PQS 2023–24, where pharmacy teams were asked to use this resource after implementing the findings and recommendations from the report.^21^

### Data analysis

Descriptive analysis was conducted by using R statistical software version 4.2.1 (R Foundation for Statistical Computing) and the results were reported as frequencies and percentages.

### Ethics

This study is a service evaluation and therefore did not require ethical approval.^17^ Further institutional ethical approval was not required, as confirmed through the UKHSA Research Ethics and Governance Group and the NHS Health Research Authority Decision tool. No patient identifiable data were recorded within the data collection. All files were handled in accordance with the Data Protection Act 2018 and the UK General Data Protection Regulation (GDPR). All community pharmacies consented to participate through the online portal.

## Results

**Figure 2.**
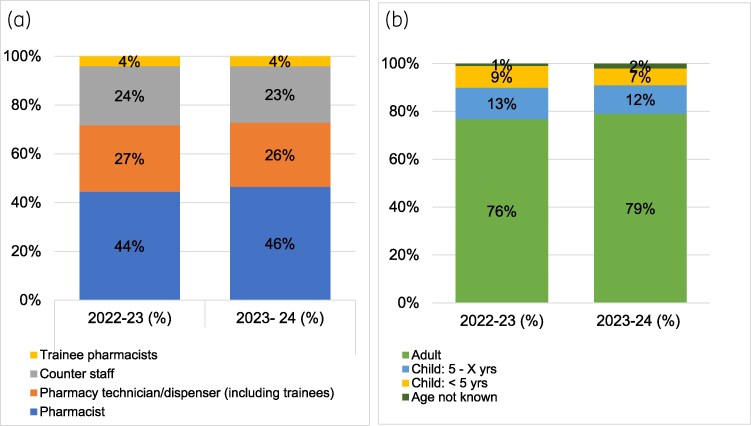
Community pharmacy consultations for an RTI during the 2022–23 and 2023–24 PQSs. (a) Pharmacy staff role managing the patient; (b) RTI consults by patient age.

A total of 225 615 patients were reviewed using the TARGET TYI-RTI leaflets over the 2 years, with 7525 community pharmacies participating in either PQS scheme, and 7407 community pharmacies participating in both PQS schemes. A total of 115 094 TARGET TYI-RTI leaflets were submitted in 2022–23 and 110 521 in 2023–24. Assessment using the TARGET TYI-RTI leaflet was mainly via the pharmacist in both years [44% (50 799) in 2022–23, and 46% (51 373) in 2023–24], followed by the pharmacy technicians/dispensers [27% (31 609) in 2022–23, and 26% (28 500) in 2023–24], counter staff and trainee pharmacists [4% (4774) in 2022–23, and 4% (4655) in 2023–24] (Figure 2). Most patients being assessed were adults in both years [76% (87 822) in 2022–23, and 79% (86 933) in 2023–24], with children over 5 years accounting for 13% (15 123) in 2022–23, and 12% (13 691) in 2023–24, and children under 5 years accounting for 9% (10 775) in 2022–23, and 7% (8137) in 2023–24] (Figure 2).

The most frequently suspected infections were similar for both years, with cough and bronchitis being the most common symptoms [33% (45 822) in 2022–23, and 31% (38 848) in 2023–24], followed by sore throat, [25% (34 220) in 2022–23, and 25% (31 099) in 2023–24] and the common cold [22% (29 802) in 2022–23, and 23% (28 627) in 2023–24] (Table 1). COVID-19 infections were one of the lowest types of infection for both years with only 1% (1587) infections in 2022–23, and 1% (1531) in 2023–24.

**Table 1.** Types of suspected infections

Suspected infections	Number of patients^a^ (%), 2022–23	Number of patients^a^ (%), 2023–24
Cough or bronchitis	45 822 (33)	38 848 (31)
Sore throat	34 220 (25)	31 099 (25)
Common cold	29 802 (22)	28 627 (23)
Sinusitis	14 538 (10.5)	15 331 (12)
Middle ear infection	9596 (7)	9012 (7)
COVID-19	1587 (1)	1531 (1)
Other	2091 (1.5)	1453 (1)
Total number of suspected infections	137 656 (100)	125 901 (100)
Total number of patients	115 094	110 521

^a^Some patients presented with multiple suspected infections—137 656 suspected infections were presented by 115 094 patients in 2022–23, and 125 901 suspected infections were presented by 110 521 patients in 2023–24.

Forty-three percent (49 813) of patients in 2022–23, and 39% (42 731) in 2023–24 were seen and managed by community pharmacy teams, requiring no escalation to the pharmacist. The pharmacists dealt with more patients in 2023–24 (46%; 51 373) compared with 2022–23 (34%; 38 603). There was a decline in the number of patients requiring escalation to the pharmacist from 23% (26 678) in 2022–23 to 15% (16 417) in 2023–24 (Table 2).

**Table 2.** Escalation of patients within pharmacy and their symptoms

	2022–23						2023–24					
Condition	Escalation within the pharmacy	Under 5 years (%)	Over 5 years (%)	Adult (%)	Age unknown (%)	Total (%)	Escalation within the pharmacy	Under 5 years (%)	Over 5 years (%)	Adult (%)	Age unknown (%)	Total (%)
Cough or bronchitis	No—escalation was not needed	1142 (1)	2115 (2)	15 998 (14)	217 (0)	19 489 (17)	No—escalation was not needed	771 (1)	1724 (2)	13 193 (12)	280 (0)	15 982 (14)
	N/A—the pharmacist was the person who spoke to the patient about their symptoms	1727 (2)	1785 (2)	12 025 (10)	113 (0)	15 663 (14)	N/A—the pharmacist was the person who spoke to the patient about their symptoms	1535 (1)	1902 (2)	13 382 (12)	60 (0)	16 894 (15)
	Yes	1522 (1)	1620 (1)	7466 (6)	92 (0)	10 709 (9)	Yes	771 (1)	955 (1)	4104 (4)	171 (0)	6006 (5)
	Total	4391 (4)	5520 (5)	35 489 (31)	422 (0)	45 861 (40)	Total	3077 (3)	4581 (4)	30 679 (28)	511 (0)	38 883 (35)
Sore throat	No—escalation was not needed	508 (0)	1809 (2)	12 547 (11)	257 (0)	15 134 (13)	No—escalation was not needed	248 (0)	1177 (1)	10 546 (10)	316 (0)	12 298 (11)
	N/A—the pharmacist was the person who spoke to the patient about their symptoms	1028 (1)	1845 (2)	8577 (7)	109 (0)	11 569 (10)	N/A—the pharmacist was the person who spoke to the patient about their symptoms	677 (1)	2229 (2)	11 778 (11)	157 (0)	14 854 (13)
	Yes	908 (1)	1698 (1)	4855 (4)	79 (0)	7546 (7)	Yes	325 (0)	908 (1)	2686 (2)	52 (0)	3975 (4)
	Total	2444 (2)	5352 (5)	25 979 (23)	445 (0)	34 249 (30)	Total	1250 (1)	4314 (4)	25 010 (23)	525 (0)	31 127 (28)
Common cold	No—escalation was not needed	1047 (1)	1984 (2)	12 041 (10)	218 (0)	15 303 (13)	No—escalation was not needed	721 (1)	1569 (1)	10 641 (10)	271 (0)	13 214 (12)
	N/A—the pharmacist was the person who spoke to the patient about their symptoms	1292 (1)	1321 (1)	6108 (5)	74 (0)	8803 (8)	N/A—the pharmacist was the person who spoke to the patient about their symptoms	1387 (1)	1712 (2)	8730 (8)	142 (0)	11 982 (11)
	Yes	1127 (1)	1210 (1)	3324 (3)	56 (0)	5722 (5)	Yes	580 (1)	740 (1)	2095 (2)	39 (0)	3457 (3)
	Total	3466 (3)	4515 (4)	21 473 (19)	348 (0)	29 828 (26)	Total	2688 (2)	4021 (4)	21 466 (19)	452 (0)	28 652 (26)
Sinusitis	No—escalation was not needed	74 (0)	184 (0)	5446 (5)	85 (0)	5794 (5)	No—escalation was not needed	44 (0)	119 (0)	4870 (4)	123 (0)	5161 (5)
	N/A—the pharmacist was the person who spoke to the patient about their symptoms	127 (0)	191 (0)	4679 (4)	51 (0)	5052 (4)	N/A—the pharmacist was the person who spoke to the patient about their symptoms	114 (0)	265 (0)	7131 (6)	78 (0)	7595 (7)
	Yes	116 (0)	182 (0)	3355 (3)	48 (0)	3704 (3)	Yes	77 (0)	109 (0)	2360 (2)	41 (0)	2589 (2)
	Total	317 (0)	557 (0)	13 480 (12)	184 (0)	14 550 (13)	Total	235 (0)	493 (0)	14 361 (13)	242 (0)	15 345 (14)
Middle ear infection	No—escalation was not needed	190 (0)	289 (0)	1268 (1)	34 (0)	1783 (2)	No—escalation was not needed	97 (0)	147 (0)	882 (1)	30 (0)	1157 (1)
	N/A—the pharmacist was the person who spoke to the patient about their symptoms	699 (1)	746 (1)	2307 (2)	36 (0)	3791 (3)	N/A—the pharmacist was the person who spoke to the patient about their symptoms	1035 (1)	1220 (1)	2892 (3)	78 (0)	5230 (5)
	Yes	758 (1)	865 (1)	2352 (2)	52 (0)	4030 (4)	Yes	463 (0)	603 (1)	1519 (1)	46 (0)	2633 (2)
	Total	1647 (1)	1900 (2)	5927 (5)	122 (0)	9604 (8)	Total	1595 (1)	1970 (2)	5293 (5)	154 (0)	9020 (8)
COVID-19	No—escalation was not needed	11 (0)	20 (0)	524 (0)	13 (0)	568 (0)	No—escalation was not needed	4 (4)	13 (0)	483 (0)	20 (0)	520 (0)
	N/A—the pharmacist was the person who spoke to the patient about their symptoms	20 (0)	16 (0)	549 (0)	14 (0)	600 (1)	N/A—the pharmacist was the person who spoke to the patient about their symptoms	14 (0)	34 (0)	659 (0)	17 (0)	725 (1)
	Yes	16 (0)	22 (0)	375 (0)	7 (0)	420 (0)	Yes	6 (0)	18 (0)	258 (0)	5 (0)	287 (0)
	Total	47 (0)	58 (0)	1448 (1)	34 (0)	1588 (1)	Total	24 (0)	65 (0)	1400 (1)	42 (0)	1532 (1)
Other	No—escalation was not needed	39 (0)	45 (0)	560 (0)	12 (0)	657 (1)	No—escalation was not needed	19 (0)	29 (0)	352 (0)	23 (0)	423 (1)
	N/A—the pharmacist was the person who spoke to the patient about their symptoms	114 (0)	86 (0)	546 (0)	16 (0)	763 (1)	N/A—the pharmacist was the person who spoke to the patient about their symptoms	68 (0)	67 (0)	533 (0)	25 (0)	694 (1)
	Yes	125 (0)	106 (0)	430 (0)	12 (0)	674 (1)	Yes	25 (0)	28 (0)	278 (0)	6 (0)	337 (1)
	Total	278 (0)	237 (0)	1536 (1)	40 (0)	2093 (2)	Total	112 (0)	124 (0)	1163 (1)	54 (0)	1454 (1)
Total	No—escalation was not needed	2659 (0)	5505 (5)	40 915 (36)	734 (1)	49 813 (43)	No—escalation was not needed	1731 (2)	4198 (4)	35 863 (32)	939 (1)	42 731 (39)
	N/A—the pharmacist was the person who spoke to the patient about their symptoms	4257 (4)	4965 (4)	29 028 (25)	353 (0)	38 603 (34)	N/A—the pharmacist was the person who spoke to the patient about their symptoms	4389 (4)	6574 (6)	39 804 (36)	606 (1)	51 373 (46)
	Yes	3859 (39	4653 (4)	17 879 (16)	287 (0)	26 678 (23)	Yes	2017 (2)	2919 (3)	11 266 (10)	215 (0)	16 417 (15)
	Total	10 775 (9)	15 123 (13)	87 822 (76)	1374 (1)	115 094 (100)	Total	8137 (7)	13 691 (12)	86 933 (79)	1760 (2)	110 521 (100)

From these consultations, 12% (13 248) of patients were signposted to another healthcare provider in 2022–23, and 7% (8250) in 2023–24, of which 6% (6859) required immediate escalation in 2022–23, and 4% (4075) in 2023–24 (Table 3). The most common signposting destination was the patient’s GP [5% (5507) in 2022–23, and 3% (3333) in 2023–24] (Table 4).

**Table 3.** Escalation of patients outside of community pharmacy

Symptoms requiring further escalation outside of community pharmacy	Number of patients (%) 2022–23	Number of patients (%) 2023–24	*P* value
No	101 846 (88)	102 271 (93)	<0.001
Yes	13 248 (12)	8250 (7)	<0.001
Total	115 094 (100)	110 521 (100)	

**Table 4.** Signposting and escalation destination

	Number of urgent referrals (%), 2022–23	Number of urgent referrals (%), 2023–24	*P* value
Signposted			
Immediately	6859 (6)	4075 (4)	<0.001
If symptoms did not improve within 48 h	3082 (3)	2044 (2)	<0.001
If symptoms got worse	2929 (3)	1905 (2)	<0.001
N/A (not referred to other services)	378 (0)	226 (0)	<0.001
Total number of patients	115 094	110 521	
Escalation destination			
Yes—GP	5507 (5)	3333 (3)	<0.001
Yes—out-of-hours/NHS 111 service	980 (1)	504 (0)	<0.001
Yes—Accident and Emergency	280 (0)	177 (0)	<0.001
Yes—other	92 (0.08)	61 (0.06)	<0.05
Total number of patients	115 094	110 521	

The most common types of reason for escalation identified for immediate referral were ‘symptoms getting worse’ [42% (3927) in 2022–23, and (47%) 2511 in 2023–24] and other (non-specified symptoms) [18% (1682) in 2022–23, and 18% (974) in 2023–24] (Table 5). Confusion, being very drowsy or having slurred speech was one of the least common symptoms in both years [2% (142) in 2022–23, and 1% (49) in 2023–24]. Passing little to no urine was the least common escalation symptom [1% (108) patients in 2022–23, and 1% (68) in 2023–24].

**Table 5.** Type of escalation symptoms identified for immediate referral

Type of escalation symptoms identified for immediate referral	Number of symptoms (%), 2022–23	Number of symptoms (%), 2023–24	*P* value
Symptoms are getting worse	3927 (42)	2511 (47)	<0.001
Difficulty breathing	953 (10)	455 (9)	<0.001
Difficulty swallowing or are drooling	834 (9)	422 (8)	<0.001
Chest pains	684 (7)	395 (7)	<0.001
Severe headache and vomiting	318 (3)	139 (3)	<0.001
Skin is very cold	324 (4)	95 (2)	<0.001
Coughing up blood	289 (3)	218 (4)	<0.001
Confusion, very drowsy or have slurred speech	142 (2)	49 (1)	<0.001
Passed little to no urine	108 (1)	68 (1)	<0.001
Other (non-specified symptoms)	1682 (18)	974 (18)	<0.001
Total	9261 (100)	5326 (100)	

Most patients were offered OTC remedies [85% (97 551) in 2022–23, and 89% (98 343) in 2023–24], of which the majority accepted [80% (91 480) in 2022–23, and 84% (93 057) in 2023–24]. There was a decline in the number of patients who were not offered any OTC remedies [15% (17 543) in 2022–23 to 11% (12 178) in 2023–24] (Table 6). The most common OTC remedies were pain relief medication, expectorant cough syrups, throat lozenges and oral decongestants in both years. In contrast, nasal sprays and all-in-one preparations were the least commonly offered OTC remedies.

**Table 6.** OTC supply and type of OTC supplied

OTC medicines offered to patient	Consultations (%), 2022–23	Consultations (%), 2023–24	*P* value
Yes—supplied	91 480 (80)	93 057 (84)	<0.001
Yes—declined	6071 (5)	5286 (5)	<0.001
No	17 543 (15)	12 178 (11)	<0.001
Total	115 094 (100)	110 521 (100)	

Type of OTC medicine supplied	Number of OTC medicines (% of people who were given OTC recommendations), 2022–23	Number of OTC medicines (% people who were given OTC recommendations), 2023–24	
Pain relief	38 818 (40)	37 576 (38)	<0.001
Cough medicine—expectorant	25 263 (26)	22 438 (23)	<0.001
Anaesthetic throat spray	18 511 (19)	17 408 (18)	<0.001
Throat lozenges	16 575 (17)	15 313 (16)	<0.001
Oral decongestant	16 559 (17)	16 317 (17)	<0.001
Cough medicine—suppressant	13 051 (13)	13 462 (14)	<0.05
Nasal spray	9793 (10)	9881 (10)	0.9553
All-in-one cold and flu preparations	938 (1)	— (0)	
Prescription-only medicine by patient group direction	—	3255 (3)	
Other	7166 (7)	9458 (10)	<0.001
Total	97 551 (100)	98 343 (100)	

Self-care advice was given to 95% (109 474) of patients in 2022–23, with 31% (35 624) receiving additional patient information leaflets, and 97% (106 770) in 2023–24, of whom 34 242 (31%) received additional patient information leaflets (Table 7).

**Table 7.** Self-care advice and the supply of information leaflets

Self-care advice given	2022–23	2023–24	*P* value
Yes—verbal advice only provided	73 850 (64)	72 528 (65)	<0.001
Yes—verbal advice and patient information leaflets provided	35 624 (31)	34 242 (31)	0.8801
No	5620 (5)	3751 (3)	<0.001
Total	115 094 (100)	110 521 (100)	

## Discussion

### Use of the TARGET TYI-RTI leaflets and escalations

Most patients were managed within the community pharmacy, with a significant proportion being managed by the extended community pharmacy team, (technicians and counter staff), which increased from 2022–23 to 2023–24. Further, fewer patients in 2023–24 required escalation outside the community pharmacy to other healthcare providers such as GPs. Evidence from the principal findings of a cohort study showed that it is very unlikely that patients would develop pneumonia within 7 days of an RTI illness against predictive indicators such as pulse, temperature, chest crackles and O_2_ saturation levels, and therefore would not require a course of antibiotics.^23^ Patient reassurance is supported by the TARGET TYI-RTI leaflet, which explains which symptoms require escalation, as well as reassuring patients about the trajectory of current ones.^24^ These findings agree with a randomized controlled trial (RCT) confirming that the use of the leaflet was associated with a lower likelihood of referrals to GPs for certain RTIs (*P* < 0.05) and more frequent provision of self-care advice than the control group (*P* = 0.06).^14^

As part of historic PQS, community pharmacists have also received red flag and sepsis training, and are therefore able to assess patients’ symptoms so they can receive the similar/same assessment that they would have received with the GP or an urgent care centre (UCC) setting.^25^ An explanation for the reduction in the referrals could be the launch of the Community Pharmacy—Pharmacy First service. This service was launched on 31 January 2024, where patients in England can receive treatment (and, if appropriate, antibiotics) for seven common conditions including sinusitis, sore throat and ear infection.^26^

Antibiotics are often misused because they are thought of as ‘benign’ drugs.^27^ Primary care is a key component of a high-performing national healthcare system, where in England, the majority of antibiotics are prescribed.^28^ It is therefore important that the public is regularly educated to push positive behaviours regarding antibiotic use and to promote AMS. The TARGET TYI-RTI leaflets allow structured conversations with patients specific to the most common ailment—RTI. It is also important for healthcare professionals, particularly community pharmacy teams, to be aware of the latest evidence-based research, which can be applied practically to counsel their patients. The TARGET TYI-RTI leaflets have aided in England’s community pharmacies counselling and educating patients about their symptoms, helping to safety-net and reassure patients, as well as escalate where appropriate. This in turn can help with reducing inappropriate GP appointments and hospital admissions, and prevent the inappropriate use of emergency departments. The use of incentivization via the PQS has allowed significant clinical progress in and awareness of AMS to be made at pace for all of England’s community pharmacies. It is not clear if such progress could be achieved in any other way.

### Self-care, lifestyle advice and benefit from the use of nasal sprays

The majority of patients were offered and supplied with OTC remedies, which showed a statistically significant increase in 2023–24 (84%; 93 057) (*P* < 0.001). Although only 10% of those who received OTC medicines were given nasal sprays, a recent RCT demonstrated that nasal sprays reduce the duration of illness and the need for antibiotics for URTI symptoms (including coughs, colds, sore throat, sinus or ear infections, influenza or COVID-19).^29^ Evidence suggests that nasal sprays containing carrageenan reduce both the duration and severity of symptoms, whilst saline sprays are thought to reduce the viral load.^30,31^ This is a scalable intervention that has an important and practical role in managing the public during NHS peak times whilst promoting AMS principles.^32^ This intervention is suitable for adults, including those over 65 years of age, with comorbidities (such as heart disease, asthma, lung disease, diabetes, stroke and obesity). Community pharmacy teams should promote this evidence to patients when counselling them. In addition, amendments to the TARGET toolkit resources could also help remind both patients and healthcare professionals about the use of this intervention. Further counselling could promote the use of physical activity (where applicable) and stress management, with the same study showing a reduction in the severity, incidence of symptoms and the use of antibiotics.^29^

## Strengths and limitations

Just under a quarter of a million patients were reviewed using the TARGET TYI-RTI leaflets over the 2 years of the PQS, with nearly 7500 community pharmacies participating in both years. Much like similar PQS studies, it is recognized that community pharmacy teams are likely to behave differently when they are incentivized.^16,17,33–35^ Further, there was a decline in participation in the scheme due to the lack of capacity and financial investment.^36^ Further studies could involve extrapolating the use of the TARGET TYI-RTI leaflets with the launch of the Pharmacy First Service (31 January 2024), where community pharmacy is now able to treat patients with antibiotics, if required, for common respiratory infections including sore throat, sinusitis and acute otitis media.^36^

### Conclusions

Community pharmacy teams have successfully used the TARGET TYI-RTI leaflets to manage the large number of patients providing self-care and reassurance, as well as safety-netting and when to reconsult a healthcare provider. A significant number of these patients were managed by the wider community pharmacy team and did not require escalation to the pharmacist. Further, few patients required escalation to other healthcare providers and were suitably managed by community pharmacy teams. Implementing the TARGET TYI RTI leaflet—a primary care intervention (which was originally designed for GPs) into existing community pharmacy infrastructure proved scalable, demonstrating its national (and international) potential for AMS impact. There has been significant improvement in AMS within the community pharmacy sector, which has been driven by financial incentivization from the pharmacy quality scheme.

## Data Availability

The data presented in this study are available upon reasonable request from the corresponding author.
